# A Data-Driven Approach for Estimating Type 2 Diabetes-Related Costs in Greece

**DOI:** 10.3390/jmahp13040053

**Published:** 2025-10-15

**Authors:** Elisavet Nika, Thomas Tsiampalis, Athanasios Sachlas, Evangelos Liberopoulos, Sotirios Bersimis, Dimitrios Georgakellos

**Affiliations:** 1 Department of Business Administration, University of Piraeus, 80 Karaoli & Dimitriou Str., 18534 Piraeus, Greece; sbersim@unipi.gr (S.B.); dgeorg@unipi.gr (D.G.); 2 Department of Nutrition and Dietetics, Harokopio University, El. Venizelou 70, 17676 Athens, Greece; ttsiampalis@uth.gr; 3 Department of Nutrition and Dietetics, University of Thessaly, Argonafton 1C, 42132 Trikala, Greece; 4 Department of Computer Science and Biomedical Informatics, University of Thessaly, Papasiopoulou 2-4, 35131 Lamia, Greece; asachlas@uth.gr; 5 1st Department of Internal Medicine, School of Medicine, National and Kapodistran University of Athens, 17 Agiou Thoma Str., 11527 Athens, Greece; elibero@med.uoa.gr

**Keywords:** aging effect, budgets’ optimization strategy, healthcare budgets, meta-analysis, type 2 diabetes

## Abstract

Type 2 diabetes (T2D) constitutes a major health problem, reaching alarming rates over the last decades, especially due to contemporary lifestyle and associated obesogenic environments, as well as the aging population. Diabetes not only causes social consequences but also leads to increasing healthcare costs, posing a significant challenge for the health system. This paper applies a five-step approach for estimating T2D-related costs in Greece. The approach initially estimates the T2D-related ICD10 prevalence and the target population. Next it applies the appropriate therapeutic protocols to identify the most appropriate treatments. Subsequently, it calculates the total cost of medical treatments for each target population, based on the distribution of patients between the different treatments and treatment lines. Finally, based on the diagnostic and treatment protocols, it calculates the annual direct costs associated with the cost categories. Using the estimated future population of the country, the proposed methodology can also project the budget required, under certain conditions, to deal with T2D. The analysis estimated that T2D-related costs in 2021 under rational use of resources were EUR 1,397,871,172.55 billion and EUR 1,512,934,947.63 billion projected in the year 2030 considering the aging effect, per cost category, and in total, presenting an increase of approximately 115 million euros in 2030 compared to 2021. The term “rational use of resources” in this study refers to the use of internationally recognized, evidence-based diagnostic and therapeutic protocols, as adopted by the Greek Ministry of Health. This scenario represents an idealized standard of care rather than actual real-world adherence and is used to estimate the potential resource needs under optimal medical practice conditions. An inflation rate of 4.2% was applied to costs between 2021 and 2030. The analysis showed that the highest percentage (39%) of the total T2D-related healthcare expenditures is associated with complications that occur in T2D patients. Despite a comparatively modest prevalence of T2D in Greece relative to other European and Mediterranean countries, the economic burden associated with its management remains high. The aging of the population will lead to an increase in the total cost of T2D. The applied methodology of estimating budgets by aggregating categories of expenses under a specific disease (ICD10), instead of dividing budgets into categories of expenses, can successfully lead to the optimization and rationalization of expenses according to actual needs. The findings underline the significant economic burden of T2D in Greece, particularly due to complications and population aging. These results emphasize the urgent need for health policy strategies focusing on prevention, early intervention, and the efficient allocation of healthcare resources. The methodology applied can serve as a decision-making tool for forecasting healthcare budgets and optimizing expenditures under different population and treatment scenarios.

## 1. Introduction

Type 2 diabetes (T2D) constitutes a major health problem, which has reached alarming levels in recent decades. Type 2 diabetes (T2D) constitutes a major health problem globally and nationally, representing both a significant clinical challenge and a substantial financial burden for healthcare systems. Its prevalence continues to rise worldwide, while the costs associated with its management weigh heavily on public budgets and household expenditure.

### 1.1. Global and National Burden of T2D

Based on the latest statistics, more than 500 million people worldwide are currently living with diabetes [1]. Projections from the International Diabetes Federation (IDF) suggest that this number will surpass 600 million by 2045 [2], mainly due to population aging, rapid urbanization, and the prevalence of obesogenic environments. Simultaneously, the Global Burden of Disease project anticipates that the number of people with diabetes will reach 1.31 billion by 2050, with most cases being type 2 diabetes [3]. In addition, aging of the population and fertility rates aggravate the negative impact of health expenditures on economic growth [4]. In Greece, among adults aged 20 to 79 years, it was estimated that there were 736,100 diabetes cases in 2021, a number that is expected to increase to 783,200 cases by 2045 [5]. At the same time, several European studies report that diabetes, which ranks among the top 10 causes of death in adults, is associated with significant acute and long-term health complications [6].

### 1.2. Economic Impact of T2D

According to the latest global estimates from the IDF [7], the economic burden of diabetes is expected to increase to USD 825 billion by 2030 and USD 845 billion by 2045. Its high incidence puts significant financial pressure on healthcare systems, as 12% of the global health budget (USD 727 billion) is spent on treatment [8,9].

The management of T2D is complex, requiring a holistic approach that addresses both acute and long-term complications and leads to increased use of health resources, particularly laboratory tests and hospitalizations [10]. Mata-Cases et al. [11] revealed that higher costs for T2D patients compared to non-diabetic subjects in Spain were mainly due to hospitalizations and medications, with costs higher among patients with poor glycemic control and macrovascular complications. It is estimated that more than 45% of diabetes treatment expenditure relates to complications [12], while half of people with T2D have at least one comorbidity [8].

### 1.3. Complications, Aging, and Health System Strain

The complexity of treating T2D, compounded by multiple comorbidities, is associated with increased use of health services and polypharmacy [13]. Both the health and economic burden are expected to grow as the population ages, with approximately 60% of diabetes-related healthcare costs incurred by people over 65 years of age [14,15]. Aging is recognized as a major contributor to the diabetes epidemic, as older adults often have multiple coexisting medical conditions, which impact effective clinical management [16].

Geyer et al. [17] estimated an aggregate increase in the health costs of about 7.7 million euros for women born in 1952, amounting to less than 2% of the positive fiscal effects of the pension reform. Andrade et al. [18] highlighted the role of outpatient care in preventing complications related to chronic conditions such as diabetes.

### 1.4. The Greek Healthcare System, Cost-Containment Mechanisms, and T2D Expenditure

In Greece, the healthcare system is based on universal coverage and is financed through a combination of general taxation, social insurance contributions, and private spending. The main public health insurance fund (EOPYY) provides access to services delivered by both public and contracted private providers. The system employs various mechanisms, including clawback and rebate systems, to manage costs and ensure sustainable access to medications [19]. The clawback mechanism requires healthcare services and medication providers to reimburse the state when total expenditure exceeds predefined thresholds, while rebate mechanisms impose volume discounts or rebates on total expenditure, leveraging the healthcare system’s purchasing power [20,21,22].

Both mechanisms encourage cost-effective prescribing, enhance negotiations with pharmaceutical manufacturers, and help maintain the financial sustainability of the system. However, careful monitoring is required to balance cost control with patient access to essential medications [23,24].

Recently, Nika et al. [25] proposed a five-step methodological framework for estimating, forecasting, and monitoring healthcare costs associated with specific ICD10 codes, integrating quantitative and epidemiological data with principles of appropriate healthcare utilization.

### 1.5. Study Rationale and Objectives

Given the significant clinical and financial burden of T2D, anticipating costs for its management is essential. To our knowledge, this is among the first studies in Greece to estimate the total cost of diabetes. This study also estimates costs under the assumption of rational resource use, following internationally recognized good medical practices and protocols. Accordingly, we apply the data-driven methodology of Nika et al. [25] to estimate T2D-related expenditures in Greece, based on current diagnostic and therapeutic protocols, and project these costs to 2030 under assumptions of natural population aging.

## 2. Material and Methods

Diabetes-related health expenditures for 2021 and projections to 2030 were estimated using an aggregation instead of a division strategy, and it was based on the inputs given in Section 2.2.

### 2.1. Application Steps of the Method

**Figure 1:**
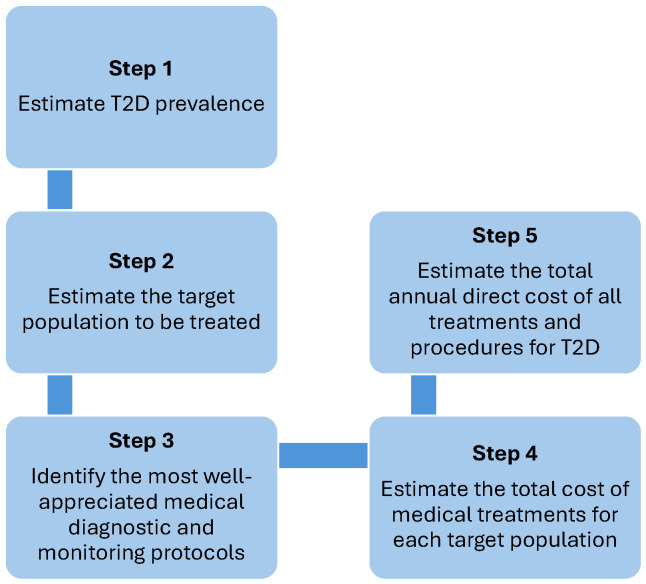
The proposed framework for estimating the type 2 diabetes-related healthcare expenditures.

Figure 1 illustrates the structure of the methodological framework. The suggested approach is divided into five main steps:

**Step 1:** Estimate the prevalence of ICD-10 codes related to type 2 diabetes, either through an appropriate sampling method or by conducting a meta-analysis of data from the literature, and calculate the size of the target population requiring treatment in Greece for the years 2021 and 2030, both overall and stratified by age groups (0–9, 10–19, 20–24, 25–49, 50–69, 70+).
**Step 2:** Calculate the target population on the basis of census figures or other reliable and validated demographic sources.
**Step 3:** Identify the criteria and conditions for initiating drug treatment, the most appropriate treatments, and the strategy for implementing optimal disease management according to the most well-appreciated therapeutic protocols.
**Step 4:** Estimate the total cost of medical treatments for each target population, based on the allocation of patients to various treatment options and therapy lines. The distribution of patients between the different treatments and treatment lines can be found by searching PubMed and Google Scholar for randomized clinical trials published in the last 5 years (or any other period the investigators decide).
**Step 5:** In line with the established diagnostic and treatment protocols (identified in Step 3), calculate the annual direct costs associated with the cost categories presented above.

More specifically, the initial stage of the model involves estimating the prevalence *p* of type 2 diabetes. Based on this, the number of affected individuals is derived as T=N×p, where *N* corresponds to the total population. Subsequently, using therapeutic protocols, the most common treatment options are identified. Assuming, for instance, that two therapies dominate, the corresponding subpopulations are calculated as $T_{1}=N\times p\times d_{1}$ and $T_{2}=N\times p\times d_{2}$, with $d_{i}$ representing the share of patients receiving treatment *i*. The costs of these treatments are then incorporated, denoted by $C_{1}$ and $C_{2}$, respectively. The overall expenditure (TC) related to T2D can thus be expressed as:

$$TC=TC_{1}+TC_{2}=N\times p\times d_{1}\times C_{1}+N\times p\times d_{2}\times C_{2}.$$

This structured five-step framework extends the model of Nika et al. [25], designed for cost estimation and long-term forecasting of ICD10-coded disease burdens. Its main advantage lies in combining epidemiological prevalence, therapeutic protocols, and reimbursement cost schemes, enabling a data-driven and policy-oriented projection of healthcare expenditures.

### 2.2. Inputs of the Method

**Input 1: Prevalence of T2D.** Evidence concerning the prevalence of T2D, both in total, as well as stratified by the population age (0–9 years, 10–19 years, 20–24 years, 25–49 years, 50–69 years, 70+ years) were drawn from the Global Burden of Disease (GBD) project, which provides a comprehensive picture of mortality and disability across countries, time, age, and sex. The GBD project, with more than 400 scientific publications since 2010, has been collected and analyzed by a consortium of more than 9000 researchers in 162 countries and territories, with the data capturing premature death and disability from 370 diseases and injuries in 204 countries and territories, by age and sex, from 1990 to the present [3].

Except for the above-mentioned source for the prevalence of T2D, we also conducted a sensitivity analysis by performing a meta-analysis. Specifically, we performed a literature search using the MEDLINE, Embase, and PubMed databases. Since we aimed to estimate the most current health expenditures related to T2D, the search focused on data from 2019 to 2025. The search concerned adult patients in Greece. Systematic searches were conducted for Greece and other Mediterranean countries, using the following search strategy: (i) Greece combined with “diabetes” AND (“prevalence” OR “incidence”); AND (ii) “insulin-dependent diabetes mellitus” OR “Type 2 diabetes”, combined with “prevalence” OR “incidence”.

In addition, relevant citations from each article were also obtained. Afterward, we conducted a random-effects meta-analysis to estimate the overall pooled prevalence of T2D. We assessed heterogeneity between studies through Cochran’s *Q* test and $I^{2}$ index, while we assessed publication bias qualitatively via visual inspection of the funnel plot and quantitatively through Egger’s linear regression test.

**Input 2: Greek population.** Data regarding the Greek (GR) population in 2021 were drawn from the 2021 Greek Population and Housing Census (https://elstat-outsourcers.statistics.gr/Census2022_GR.pdf), assessed on 20 December 2024. Data for 2030 were drawn from PopulationPyramid.net (https://www.populationpyramid.net/greece), assessed on 20 December 2024, which is based on the estimates provided by the United Nations, Department of Economic and Social Affairs, Population Division [26,27,28].

**Input 3: Prescription therapeutic protocols for T2D.** As regards the treatment of T2D, we based it on the Prescription Therapeutic Protocols for Diabetes Mellitus issued by the Greek Ministry of Health. According to the relevant literature, the treatment of diabetic patients begins with interventions trying to modify their lifestyle and treatment with antidiabetic drugs. However, there are multiple available lines of treatment. More specifically, first-line treatment usually begins with metformin, followed by the addition of sulfonylureas (SUs), meglitinides, alpha-glucosidase inhibitors, thiazolidinediones (TZDs), glucagon-like peptide-1 receptor agonists (GLP1-RAs), dipeptidyl peptidase-4 inhibitors (DPP-4i), sodium-glucose cotransporter 2 inhibitors (SGLT-2i), and insulin. Usually, the first-line treatment (monotherapy) is defined as taking metformin; if after approximately 3 months of metformin monotherapy, desired glycemic targets are not achieved, adding another antihyperglycemic drug is recommended. If the glycemic targets are not met with two classes of antihyperglycemic drugs, then a third drug is added. The choice of second or third medication includes long-acting GLP1 RAs in preference to basal insulin. The distribution of the T2D patients in each line of treatment was based on the study of Lee et al. [29].

The Greek Positive List that is published by the Greek Ministry of Health, containing all drugs reimbursed by the Greek Government, was used to retrieve the cost of each treatment line for T2D. This list contains all drugs reimbursed by the Greek Government. Due to the complexity of the treatment protocols and the vast number of treatment choices in each treatment line, we considered the medications with the minimum monthly cost covered by the Greek Social Security Fund (SSF).

**Input 4: Medical procedures for T2D-related complications.** The medical procedures (based on the Diagnosis-Related Group (DRG) system) applied in case of T2D-related complications, as well as the costs related to these DRGs, were retrieved from the DRG-matching application (http://kenicd.e-healthnet.gr), assessed on 18 December 2024, offered by the Medical Society of Athens in partnership with the Onassis Cardiac Surgery Center and the Ministry of Health. As in the case of T2D treatments, the costs of these medical procedures also concern those reimbursed by the Greek SSF. The percentage of T2D patients experiencing T2D-related complications was retrieved from the study of Deshpande et al. [30]. The final annual cost of T2D-related complications was estimated by multiplying the number of T2D patients with complications by the respective cost of DRGs related to specific complications.

**Input 5: Diagnostic tests for monitoring the course of the T2D patients.** The protocols published by the General Secretariat of the Ministry of Health was used to identify diagnostic tests for monitoring the course of T2D patients. Regarding their cost, it was retrieved from the appropriate relative regulation, and the appropriate relative decision issued by the Greek Ministry of Health and Social Solidarity.

As regards the number of T2D patients complying with the guidelines of the Greek Ministry of Health and the frequency with which they should be referred to the relevant medical examinations for monitoring, it was hypothesized that half of the patients monitor their health status on a frequent basis. The final annual cost of T2D-related medical examinations was estimated by multiplying the number of T2D patients complying with the guidelines by the sum of the annual cost of medical examination.

**Input 6: Visits/Consultations with physicians.** Information about how often individuals with diabetes typically visit their doctor was extracted from the recommendations provided by the Centers for Disease Control and Prevention (CDC). These guidelines suggest that diabetic patients who are successfully reaching their treatment objectives should have appointments with their physician twice a year (every 6 months), whereas those who are not meeting their treatment goals should have appointments every three months. Since the percentage of those who successfully achieve their treatment goals is unknown, in this study, we assumed that all patients visited their doctor 4 times. Under this assumption, the estimates obtained are an upper bound on the total cost. Finally, as regards their cost, when this paper was written, the minimum price per visit was EUR 10.

In all cases, to project the healthcare costs in 2030, an inflation rate of 4.2% was applied to costs between 2021 and 2030. The model assumes a constant prevalence rate and average treatment cost across age strata. While it is acknowledged that treatment intensity and complication rates may vary with age, stratified data on real-world treatment costs per age group were unavailable. Therefore, we applied an aggregated prevalence and cost estimate, as a first-order approximation. This assumption is explicitly noted in the Limitations Section.

The therapeutic protocols used in this study are based on the official Prescription Therapeutic Protocols published by the Greek Ministry of Health. Patient distribution across treatment lines was informed by two complementary sources: the international literature [29] and a sensitivity analysis using real-world prescription data from IQVIA Hellas (January–June 2023). This dual-source approach ensured both methodological robustness and alignment with local prescribing patterns. For cost estimations, we considered the minimum reimbursed prices as listed in the Greek Positive List, to reflect a conservative public-payer perspective. All treatment costs were assumed to remain constant over the projection period (2021–2030) due to the regulatory nature of price-setting in Greece.

In Table 1, all the parameters included in the estimation of the total annual cost of T2D are presented.

## 3. Results

### 3.1. Prevalence Estimates of T2D

According to the *IDF Diabetes Atlas 11th Edition 2025*, the prevalence of T2D in 2021 in adults (20–79 years) in Greece was 11.4%. Based on the Global Burden of Disease (GBD) project, the prevalence of T2D in 2021 in the general population of Greece was 8.48% (https://vizhub.healthdata.org/gbd-compare/), assessed on 05 December 2024,; the highest rate was found among people older than 70 years old (17.9%), followed by people 50–69 years old (13.24%).

A recent nationwide study involving 1000 people, in which some of the authors participated (results not yet published), found that the self-reported prevalence of T2D was 6% (95% CI: 4.61–7.66%). This result is comparable to that reported by Tentolouris et al. [31]. However, this study was not entirely focused on T2D.

**Figure 2:**
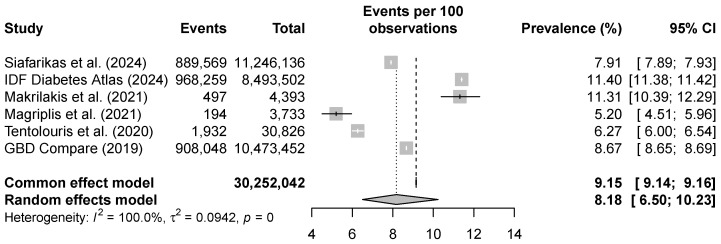
Forest plot for the T2D prevalence in Greece in the period 2019–2025. Siafarikas et al. (2024) [33]; IDF Diabetes Atlas (2024) [5]; Makrilakis et al. (2021) [34]; Magriplis et al. (2021) [35]; Tentolouris et al. (2020) [31]; GBD Compare (2019) [32].

The random-effects meta-analysis of the six most recent studies on the Greek population [5,31,32,33,34,35] yielded a pooled T2D prevalence of 8.18% (95% CI: 6.50–10.23%). High heterogeneity was observed among studies (Q=75,167.58, p<0.001 and $I^{2}=100.0\%$), justifying the use of a random-effects model. Sensitivity analyses indicated that no single study disproportionately influenced the pooled estimate, confirming the robustness of the result. Egger’s test yielded a *p*-value of 0.9175, indicating no evidence of publication bias. However, this result should be interpreted with particular caution, as only six studies were included in the meta-analysis. The prevalence estimates from individual studies alongside the overall pooled estimate are illustrated in the forest plot given in Figure 2.

### 3.2. Estimate of T2D-Related Healthcare Expenditures in 2021

As shown in Table 2, first-line treatment includes metformin monotherapy, while second-line refers to dual therapy with metformin plus another class (e.g., DPP-4i, SU). Third-line treatment indicates triple therapy, and fourth-line treatment includes combinations involving insulin. Table 3 presents these same treatment lines using alternative prevalence estimates derived from the meta-analysis. Considering the prevalence estimate given by the *IDF Diabetes Atlas 2025*, i.e., 11.4%, and considering the minimum cost associated with each treatment regimen, it became evident that the highest monthly expenses for the treatment of T2D were associated with the second line of treatment, amounting to EUR 15,284,320. This was followed by the third-line treatment, costing EUR 15,281,781, and the fourth line, which cost EUR 1,043,175. In contrast, the first line of treatment incurred the lowest monthly cost, totaling EUR 461,211. Consequently, the projected annual cost for managing T2D-related treatment regimens was estimated to be EUR 384,845,856. Here, we should note that the number of people with T2D in 2030, after considering the aging effect, was calculated according to the age-stratified prevalence of T2D based on the Global of Burden of Disease project (2019) (0–9 years: 0%; 10–19 years: 0.03%; 20–24 years: 0.68%; 25–49 years: 3.38%; 50–69 years: 13.64%, and 70+ years: 21.71%), while the distribution of patients into lines of treatment was based on Lee et al. [29], in which 31% of patients receive metformin as monotherapy and 45% receive a combination of metformin with one of the following inhibitors: (i) SUs, (ii) TZDs, (iii) DPP-4i, (iv) GLP1-RAs, (v) SGLT2i, or (vi) insulin, 23% of patients receive a triple combination of metformin and two other medications, while 1% are prescribed with a quadruple combination of the above-mentioned treatment options. Finally, 30-day cost per line of treatment per patient was considered to be fixed between 2021 and 2030.

Alternatively, when employing the prevalence estimate derived from the random-effects meta-analysis (i.e., 8.18%), as presented in Table 3, the projected annual cost for T2D-related treatment regimens was estimated to be EUR 255,975,189.49.

Regarding T2D complications, as outlined in Table 4, when factoring in the minimum monthly expenses covered by the Greek SSF, the total annual healthcare costs related to complications were projected to amount to EUR 507,957,079.

Additionally, making the hypothesis that half of the T2D patients adhered to the Greek Ministry of Health guidelines regarding the recommended frequency of medical examinations for monitoring, the healthcare expenditures associated with diagnostics were estimated to reach EUR 397,517,920. Here, we should note that the proportion of T2D patients with complications was based on the study of Deshpande et al. [30], according to which, on average, 14.06% of diabetic patients suffer from diabetic-related complications. The final annual cost of T2D-related complications was estimated by multiplying the number of T2D patients with complications by the average cost per complication. The calculation of the final annual cost of T2D-related medical examinations was based on the hypothesis that half of the T2D patients complied with the guidelines of the Greek Ministry of Health, as regards the frequency with which T2D patients should be referred to the relevant medical examinations for monitoring. The final annual cost of T2D-related medical examinations was estimated by multiplying the number of T2D patients complying with the guidelines of the Greek Ministry of Health by the sum of the annual cost per medical examination. The cost of each diabetes-related complication and medical examination was considered to be fixed between 2021 and 2030.

Utilizing the prevalence estimate derived from the random-effects meta-analysis (i.e., 8.18%), as presented in Table 5, the total annual healthcare costs tied to complications were projected to be EUR 365,923,474. In contrast, the healthcare expenses related to medical examinations were estimated at EUR 285,236,543. Hence, it appears that the largest proportion, accounting for 39% of the overall healthcare expenses associated with T2D, was attributed to complications, with medical examinations for patient monitoring making up 31%, and diabetes-related treatment constituting 30% of the total expenditures.

### 3.3. Sensitivity Analysis Based on RWD

Furthermore, using Real-World Data obtained from IQVIA Hellas, specifically concerning the sales of anti-diabetic medications in Greece from January 2023 to June 2023, we conducted a sensitivity analysis to examine how diabetic patients were distributed across various treatment lines.

According to this dataset, the first treatment line encompasses patients falling under category A10J BIGUANIDE ANTIDIABETICS, accounting for 33.4% of the patient population. Meanwhile, the second treatment line comprises categories including (a) A10H SULPHONYLUREA A-DIABS, (b) A10J BIGUANIDE ANTIDIABETICS, (c) A10K GLITAZONE ANTIDIABETICS, (d) A10L A-GLUCOSIDASE INH A-DIAB, (e) A10M GLINIDE ANTIDIABETICS, (f) A10N DPP-IV INHIBITOR A-DIABS, and (g) A10P SGLT2 INHIBITOR A-DIABS, making up 51.7% of the patient population. The third treatment line consists of patients categorized as A10S GLP-1 AGONIST A-DIABS, representing 8.8% of the patient population, while the fourth treatment line encompasses those in the A10C HUMAN INSULIN+ANALOGUES category, constituting 6.1% of the patient population.

Based on this patient distribution, the annual cost for managing treatment regimens related to T2D was estimated to be EUR 278,278,944.59 in 2021, EUR 266,299,510.15 in 2030, and EUR 281,183,016.55 in 2030, adjusting for the impact of aging. As depicted, compared to the distribution of the patients based on the article of Lee et al. [29], the total annual cost of diabetes-related treatment lines was estimated to be reduced by approximately 6%.

### 3.4. Projection of Type 2 Diabetes-Related Healthcare Expenditures in 2030

As presented in Table 1, according to PopulationPyramid.net, Greece’s total population will be reduced to 9,721,983 people in 2030, or about 3.3% compared to the country’s population in 2021. After making the assumptions presented in the Material and Methods Section, the monthly cost of treating T2D was estimated to be EUR 444,861 for the first-line treatment, EUR 14,742,458 for the second-line treatment, EUR 14,739,979 for the third-line treatment, and EUR 1,006,190 for the fourth-line treatment. This leads to an estimated annual cost for diabetes-related treatment lines equal to EUR 371,201,853, which is approximately 4% higher than the one in 2021.

At the same time, as depicted in Table 3, the same level of decrease was estimated for the complications-related costs (EUR 489,947,893 in 2030 vs. EUR 507,957,079 in 2021), as well as in the medical examinations-related costs (EUR 383,425,132 in 2030 vs. EUR 397,517,920 in 2021).

However, after considering the natural aging of the Greek population, we observe that in 2030, the cost of treating T2D will increase by approximately 59 million euros compared to the cost estimated based on the population of 2021, the complications-related cost will increase by approximately 80 million euros, and the medical examinations-related cost will increase by approximately 61 million euros.

**Figure 3:**
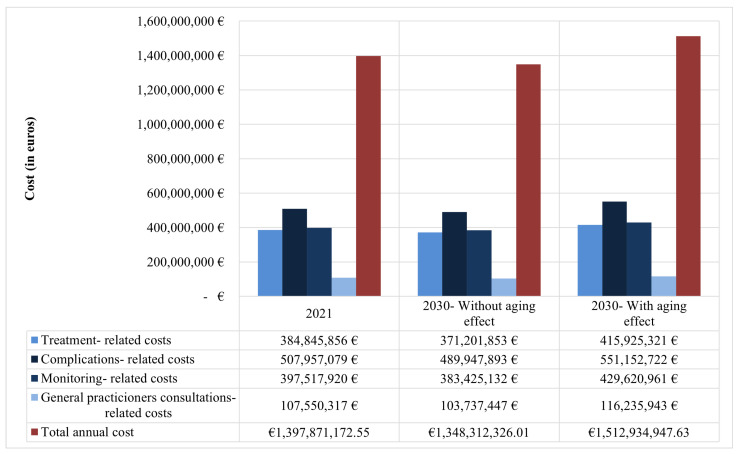
Estimate of T2D-related costs in 2021 and 2030, with and without considering the aging effect, per cost category and in total, based on the prevalence rate estimated by the *IDF Diabetes Atlas 2025*.

As presented in Figure 3, the total estimated diabetes-related cost is estimated to increase by approximately 13 million euros in 2030 after considering the population’s natural aging, compared to 2021. The estimation of general practitioners’ consultation-related costs was based on the hypothesis that on average, T2D patients have nine general practitioner consultations each year [36], as well as on the hypothesis that each consultation costs EUR 10. The number of people with T2D in 2021 and number of people with T2D in 2030 were calculated with a T2D prevalence equal to 11.4%, according to the *IDF Diabetes Atlas 2025*. The number of people with T2D in 2030, after considering the aging effect, was calculated according to the age-stratified prevalence of T2D based on the *IDF Diabetes Atlas 2025*. Annual treatment-related cost was calculated as $\sum_{i=1}^{4}\left[\left(\mathrm{Number}\;\mathrm{of}\;\mathrm{patients}\;\mathrm{in}\;\mathrm{line}\;i\right)\times \left(\text{30-day}\;\cos t\;\mathrm{for}\;\mathrm{line}\;i\right)\times 12\right]$. The percent of T2D patients with complications was based on the study of Desphande et al. [30], according to which on average 14.06% of diabetic patients suffer from diabetic-related complications. The final annual cost of T2D-related complications was estimated as (Number of T2D patients with complications) × (Average cost per complication). The calculation of the final annual cost of T2D-related medical examinations was based on the hypothesis that half of the T2D patients comply with the guidelines of the Greek Ministry of Health, as regards the frequency with which T2D patients should be submitted to the relevant medical examinations for monitoring. Final annual cost of T2D-related medical examinations was estimated as (Number of T2D patients complying with the guidelines of the GreekMinistry of Health) × (Sum of the annual cost/medical examination). The costs of medical procedures for T2D-related complications were based on the DRG system, as retrieved from the DRG-matching application (http://kenicd.e-healthnet.gr), assessed on 18 December 2024, provided by the Medical Society of Athens, in partnership with the Onassis Cardiac Surgery Center and the Greek Ministry of Health. Only costs reimbursed by the Greek Social Security Fund (SSF) were included. Protocols for monitoring T2D patients were based on guidelines from the General Secretariat of the Greek Ministry of Health (Ministerial Decision No. Γ3γ/40426, Government Gazette B’ 2221/18.07.2016). The costs of medical examinations were retrieved from the relevant regulations and decisions of the Greek Ministry of Health and Social Solidarity. It was assumed that 50% of patients complied with the recommended monitoring frequency. The total annual cost was computed as the sum of the annual cost of T2D-related complications and the annual cost of T2D-related medical examinations for monitoring. An inflation rate of 4.2% was applied to costs between 2021 and 2030, both in the 30-day cost per line of treatment per patient and T2D-related complications and T2D-related medical examinations for monitoring, as well as in the general practitioners’ consultation-related costs.

## 4. Discussion

The focus of the present work was on the most significant direct medical costs attributable to T2D, including (a) drugs being prescribed (first to fourth line of treatment) by Greek physicians in routine clinical practice for early treatment and management of T2D, (b) diagnostic tests which should be conducted on T2D patients for monitoring their health status and disease course, (c) general practitioners/physicians’ consultation expenses, as well as (d) medical procedures which T2D patients undergo regarding the treatment of complications which they may experience (e.g., diabetic complications with devastating comorbidities, based on the diagnosis-related group (DRG) system).

Even though there are other important costs as well, such as the direct non-medical costs (e.g., transportation costs) and the productivity losses and intangible costs which refer to patients’ psychological pain, discomfort, anxiety, or distress related to the disease, these were not addressed in this paper. It should be noted that these cost categories were chosen according to the American Diabetes Association, the largest components of diabetes-related medical expenditures being (i) hospital inpatient care, (ii) prescription medications to treat complications of diabetes, and (iii) anti-diabetic agents.

In summary, the study’s findings unveil significant insights into the prevalence and economic implications of T2D in Greece.

### 4.1. Prevalence of T2D

The prevalence of T2D in 2024 was notably observed at around 11.4%, with the highest rates occurring among individuals aged over 70, marking a critical trend aligned with the natural course of the disease and age-associated metabolic changes. A comprehensive meta-analysis of recent studies revealed a consolidated T2D prevalence of 8.18% (95% CI: 6.50–10.23%), acknowledging the influence of substantial heterogeneity in these estimates.

### 4.2. Cost of T2D

The economic facets of T2D were meticulously assessed, with projected annual costs for T2D-related treatment lines in 2021 varying between EUR 255,975,189.49 and EUR 384,845,856. The highest expenses were linked to second-line treatment, followed by third and fourth lines, signifying the multifaceted nature of treatment dynamics.

It is important to highlight that in 2021, total healthcare expenditure in Greece was estimated at approximately EUR 16.7 billion, corresponding to 9.2% of national GDP (EUR 182.8 billion) [37,38,39]. Of this amount, public health expenditure accounted for about EUR 10.2 billion (61.0%), while private expenditure represented roughly EUR 6.5 billion (39.0%). Within this context, T2D-related costs represented 5.6% of total healthcare expenditure and approximately 9.3% of public health spending, thereby underscoring the substantial economic burden of T2D on the Greek healthcare system.

Notably, complication-related healthcare costs ranged from EUR 365,923,474 to EUR 507,957,079, while medical examination-related expenses spanned from about EUR 285,236,543 to EUR 397,517,920. The composition of expenses was diverse, with complications accounting for 39%, medical examinations for 31%, and treatment for 30% of total T2D-related healthcare expenditures.

Considering future projections, the study’s insights into the year 2030 underscored a complex interplay of demographic shifts and aging. The anticipated 4.3% decrease in Greece’s total population would likely result in a corresponding reduction of approximately 4% in costs related to diabetes-related treatment, complications, and medical examinations. However, this moderation is offset by the natural aging of the population, as the cost of treating T2D in 2030 is projected to escalate by approximately 59 million euros compared to 2021. The burden of this increase would be particularly borne by complications and medical examination costs, expected to rise by 80 million euros and 61 million euros, respectively. Furthermore, considering general practitioners’ consultations accentuates the impact of population aging, with the total projected diabetes-related cost estimated to surge by approximately 15 million euros in 2030 when juxtaposed against 2021. Collectively, these findings illuminate the intricate relationship between T2D prevalence, economic implications, and the dynamic interplay of demographic changes, highlighting the imperative of strategic healthcare planning to address the challenges posed by T2D in the coming years.

The existing body of literature in the realm of healthcare cost estimation and the consequences of chronic diseases, including T2D, furnishes a thorough context for the methodology and outcomes outlined in this study. Numerous research endeavors have probed into the escalating global prevalence of T2D and its ramifications on healthcare expenditures. Works such as those authored by Saeedi et al. [1], Forouhi and Wareham [2], and Ong et al. [3] have supplied crucial statistics and forecasts, spotlighting the alarming surge in the number of individuals affected by T2D and the resulting financial strain. Furthermore, investigations conducted by Bommer et al. [40], Acs et al. [8], Cho et al. [9], and Williams et al. [7] have illuminated the immediate and long-term complications of T2D, underscoring its substantial influence on healthcare costs. These studies have paved the way for a deeper understanding of the significance of comprehensive cost estimation methodologies. Additionally, literature exemplified by Kalyani et al. [16] has delved into the distinct challenges presented by an aging population in the management of chronic diseases, emphasizing the necessity for customized healthcare strategies. The evolving landscape of research concerning diabetes and other persistent conditions continues to provide a robust basis for pioneering methodologies seeking to address the intricate financial aspects of healthcare amid shifting demographics and disease patterns.

### 4.3. Dynamics of Type 2 Diabetes and Aging Resilience

The findings presented here can be clarified by examining the complex interplay of factors that influence the landscape of T2D in Greece. Prevalence rates, especially among individuals over 70 years old and those between 50 and 69, reflect the natural progression of the disease and age-related changes in metabolic resilience. Specifically, the decline in biological resilience, which refers to the capacity to recover and regain vitality, is a crucial aspect of aging. This decline significantly contributes to an increased vulnerability to mortality as individuals age, a phenomenon observed in humans and other animal species [41,42]. The widespread nature of this decrease in the physiological ability to recover, coupled with the rising mortality risk as people grow older (termed the demographic characterization of aging), highlights the importance of understanding the mechanisms that underlie this diminishing resilience in the field of aging research [43,44,45].

In particular, the complex interplay of aging with T2D exacerbates the risk of complications and comorbidities, necessitating more frequent and intensive medical interventions. This increased use of healthcare, combined with the requirement for several medications and specialized treatments, significantly increases the financial burden [46]. Moreover, the aging population often experiences a higher prevalence of complications, further intensifying the need for healthcare services and elevating the associated costs [47]. Therefore, understanding the intricate mechanisms linking population aging and the rise in diabetes-related expenses is crucial for developing targeted healthcare strategies and policies to address the evolving healthcare needs of an aging society.

### 4.4. Factors Shaping Type 2 Diabetes Prevalence in Greece

The observed prevalence of T2D does not stem solely from individual aging processes; instead, it arises from a complex interplay of diverse factors influencing its emergence in the Greek population. Societal lifestyle patterns exert a central role in molding this prevalence. The adoption of contemporary lifestyles, characterized by suboptimal dietary preferences and sedentary behaviors, has seen a steady rise, potentially fostering an environment conducive to the development of T2D [48]. Urbanization serves to magnify these trends, frequently resulting in shifts in dietary practices and decreased levels of physical activity. These urban-induced alterations may impact metabolic health and, in consequence, susceptibility to T2D [49]. Genetic predispositions also factor into the equation when considering T2D prevalence. The genetic composition of the Greek population may encompass specific traits that predispose individuals to an elevated risk of T2D development [50,51]. These genetic elements interact with environmental influences, such as lifestyle and urbanization, to collectively mold the disease’s prevalence. For instance, a genetic predisposition to insulin resistance could amplify the consequences of unhealthy lifestyle choices, thereby contributing to higher T2D rates [52].

### 4.5. Economic Implications and Insights from Studies on Estimated Expenses Associated with Type 2 Diabetes

The findings of similar studies investigating the estimated expenses associated with T2D offer valuable insights into the economic impact of this prevalent chronic condition. These studies collectively highlight the substantial financial burden imposed by T2D on individuals, healthcare systems, and society as a whole [53,54,55]. A consistent thread running through these studies is the recognition that the costs of managing T2D extend far beyond the immediate medical expenditures. Direct medical costs, encompassing medications, doctor visits, hospitalizations, and diagnostic tests, constitute a significant portion of the expenses [56].

However, the impact of T2D-induced complications emerges as a pivotal contributor to the overall economic burden. The prevalence and severity of complications, such as cardiovascular issues, neuropathy, and kidney disease, significantly escalate healthcare costs, emphasizing the importance of effective prevention and management strategies [57]. The analysis of Lesniowska et al. [58] revealed that both direct and indirect costs are highly driven by the cost of diabetes complications. Furthermore, comparative analyses across different populations and healthcare systems highlight the intricate relationship between healthcare access, treatment choices, and expenditures. Disparities in access to care can translate into differing costs, as patients with limited access may face higher expenses due to late-stage interventions or emergency care [59,60].

Projection studies that anticipate future T2D-related expenses underscore the impending challenges that healthcare systems and policymakers must address. Specifically, as outlined by the CDC, numerous efficacious strategies for diabetes prevention and management demonstrate favorable cost-effectiveness when evaluated in terms of cost per quality-adjusted life year (QALY) gained. Public health interventions with costs less than USD 50,000 per QALY are broadly recognized as exhibiting strong value and being deemed cost-effective [61,62]. Moreover, projection studies highlight the dynamic nature of T2D-related expenses in response to demographic changes. Derived from the investigation by Waldeyer et al. [63], which sought to anticipate forthcoming expenses associated with T2D in Germany through the integration of demographic transitions, disease advancement, and undiagnosed instances using a time-discrete Markov model, the foreseen trajectory indicated a 79% increase in annual diabetes expenditures. These expenses were anticipated to rise from EUR 11.8 billion in 2010 to EUR 21.1 billion in 2040 (EUR 9.5 billion to EUR 17.6 billion for diagnosed cases). Conducted by bommer2018global, another study delved into modeling the economic burden of diabetes in individuals aged 20 to 79, factoring both absolute values and the proportion relative to gross domestic product (GDP). The study harnessed epidemiological, demographic, and recent GDP forecasts for 180 countries. Their findings indicated that the global economic burden would escalate, rising from U.S. USD 1.3 trillion in 2015 to USD 2.2 trillion under the baseline scenario, USD 2.5 trillion in past trend scenarios, and USD 2.1 trillion in target scenarios by 2030. This shift corresponds to an increase in costs, proportionate to global GDP, from 1.8% in 2015 to a peak of 2.2%.

Although Greece exhibits a lower prevalence of T2D compared to other European countries, per-patient healthcare costs remain relatively high. For example, average annual direct costs per T2D patient in Spain and Italy have been estimated at approximately EUR 1400–EUR 2000 [11], while our estimates suggest a comparable or higher burden in Greece. This may reflect differences in treatment patterns, reimbursement levels, or inefficiencies in care delivery and warrants further investigation.

While this study focused on the current cost structure, substantial evidence supports the cost-effectiveness of preventive strategies in reducing long-term T2D complications. Programs focusing on lifestyle interventions, early screening, and glycemic control have been shown to reduce both healthcare utilization and costs. Incorporating the long-term savings from such interventions into future projections could provide more policy-relevant insights.

### 4.6. Limitations

Due to its nature, our study has several limitations. The system of safeguarding good health is highly supervised by regulatory authorities and is decisively dependent on the decisions of these authorities. A significant change in the prices of pharmaceuticals, the strengthening of the use of generic drugs, the pricing of DRG, or changing the treatment protocols and the list of reimbursable diagnostic tests can greatly affect the budget plan of the present study. Also, the estimated health costs are in current terms as future inflation is not easy to predict.

In addition, the predictions of competent scientists for the more frequent occurrence of pandemics create scenarios of uncertainty as to the total quantity of resources required to provide adequate public health services at any given time.

We recognize that several assumptions were applied for tractability. These included constant unit costs from 2021 to 2030, fixed prevalence and treatment cost across age groups, and full adherence to clinical protocols. While simplifying, these assumptions enabled modeling under a standardized framework. Nonetheless, they are explicitly acknowledged as limitations and offer opportunities for refinement in future research.

This study focused exclusively on direct medical costs reimbursed by the Greek Social Security Fund. While these represent a substantial portion of total T2D-related expenses, indirect costs—such as productivity losses, informal caregiving, transportation, and psychological burden—were not included. Their omission limits the comprehensiveness of the total economic burden. Future research should aim to incorporate both direct and indirect costs using societal perspective models.

Finally, one might argue that the high heterogeneity observed across studies in the meta-analysis is a limitation of this study. While we employed a random-effects model to account for the variability in effect sizes, the pooled effect size should be interpreted cautiously, as it represents an average across highly diverse studies rather than a definitive estimate of effect. However, although estimating prevalence is a crucial parameter of the proposed methodology, the purpose of this manuscript was not to accurately estimate prevalence. The user of the proposed methodology can use any prevalence they consider valid and accurate.

The findings have important policy implications. Given that 39% of T2D-related costs are due to complications, investments in early detection, preventive care, and adherence support could yield substantial cost savings. The applied framework allows policymakers to simulate how demographic trends or treatment strategies impact long-term budget planning.

Future studies should integrate indirect costs and consider age-specific treatment pathways and costs. Microsimulation or Markov modeling could also allow for dynamic transition probabilities across health states. Additionally, evaluating the cost-effectiveness of preventive interventions in the Greek healthcare context would provide critical evidence for resource prioritization.

## 5. Conclusions

In this paper, we applied a five-step data-driven methodology for the estimation and projection of T2D-related costs in Greece, according to the relevant diagnostic and therapeutic protocols implemented in Greece and considering the aging effect. In conclusion, this research sheds light on the intricate landscape of T2D in Greece, offering valuable insights into its prevalence and associated economic implications. The observed prevalence rates, particularly among the elderly population and those aged 50–69, underscore the natural progression of the disease and the influence of age-related metabolic changes. A significant aspect contributing to this prevalence is the decline in biological resilience associated with aging, which heightens vulnerability to mortality. This decline, seen in humans and various animal species, underscores the need to delve deeper into the mechanisms underlying aging-related resilience waning. Furthermore, the prevalence of T2D arises from a complex interplay of various factors. Lifestyle patterns, urbanization, and genetic susceptibilities within the Greek population all contribute to the emergence of T2D. Modern lifestyles characterized by poor dietary habits and sedentary behaviors, exacerbated by urbanization, create an environment conducive to T2D development. Genetic predispositions interact with these environmental factors, amplifying the risk.

The economic burden associated with T2D is substantial and multifaceted. The estimated annual costs of T2D-related treatment and complications highlight the challenges faced by healthcare systems and individuals alike. The projection studies not only underscore the impending challenges in the future but also emphasize the potential of cost-effective prevention and management strategies, as well as the dynamic nature of expenses in response to demographic changes. Looking ahead to 2030, the projections indicate a complex interplay between population aging and cost dynamics. While a decrease in the total population might lead to a reduction in certain costs, the cost of treating T2D is set to rise due to the natural aging of the population. This necessitates strategic planning to address the evolving economic landscape of T2D management.

In summary, these findings collectively emphasize the urgent need for comprehensive strategies that consider the multidimensional nature of T2D, its interplay with aging and genetics, and the economic implications associated with its prevalence. Addressing the challenges posed by T2D requires a multifaceted approach that integrates public health interventions, personalized care, and policies tailored to the evolving needs of an aging population. 

## Figures and Tables

**Table 1:** The parameters included in the estimation of T2D’s total annual cost.

*N*	Total population
*p*	Prevalence of T2D
*T*	Number of people suffering from T2D computed as N×p
$d_{i}$	Proportion of patients who receive treatment *i*
$T_{i}$	Number of patients who receive treatment *i* computed as $T\times d_{i}$
$C_{i}$	Cost of treatment *i*
$TC_{i}$	Total cost of treatment *i* computed as $T\times d_{i}\times C_{i}$
TC	Total annual cost computed as the sum of the total costs of all treatments
$CC_{j}$	Cost of complication *j* related to T2D
TCC	Total annual cost of T2D-related complications computed as the sum of the costs of all complications
$CME_{k}$	Cost of medical examination *k* for monitoring related to T2D
TCME	Total annual cost of T2D-related medical examinations computed as the sum of the costs of all medical examinations
TCGP	Total annual cost of T2D-related general practitioners’ consultations
TAC	Total annual cost of T2D computed as TC+TCC+TCME+TCGP

**Table 2:** Estimate of total annual cost of T2D-related treatment in 2021 and 2030, with and without considering the aging effect, based on the prevalence rate estimated by the *IDF Diabetes Atlas 2025*.

Greek Population in 2021	10,482,487	Greek Population in 2030	9,721,983	Greek Population in 2030	9,721,983
**Number of People with T2D**	**1,195,004**	**Number of People with Type T2D**	**1,108,306**	**Number of People with T2D, After Considering the Aging Effect**	**1,241,837**
**Distribution of patients into lines of treatments**		**Distribution of patients into lines of treatments**		**Distribution of patients into lines of treatments**	
First line	31%	First line	31%	First line	31%
Second line	45%	Second line	45%	Second line	45%
Third line	23%	Third line	23%	Third line	23%
Fourth line	1%	Fourth line	1%	Fourth line	1%
**Number of patients in each line of treatment**		**Number of patients in each line of treatment**		**Number of patients in each line of treatment**	
First line	370,451	First line	343,575	First line	384,969
Second line	537,752	Second line	498,738	Second line	558,827
Third line	274,851	Third line	254,910	Third line	285,623
Fourth line	11,950	Fourth line	11,083	Fourth line	12,418
**30-day cost for each line of treatment**		**30-day cost for each line of treatment**		**30-day cost for each line of treatment**	
First line	EUR 461,211	First line	EUR 444,861	First line	EUR 498,458
Second line	EUR 15,284,320	Second line	EUR 14,742,458	Second line	EUR 16,518,660
Third line	EUR 15,281,781	Third line	EUR 14,739,979	Third line	EUR 16,515,935
Fourth line	EUR 1,043,175	Fourth line	EUR 1,006,190	Fourth line	EUR 1,127,390
**Total annual cost for diabetes-related treatment lines**	**EUR 384,845,856**	**Total annual cost for diabetes-related treatment lines**	**EUR 371,201,853**	**Total annual cost for diabetes-related treatment lines**	**EUR 415,925,321**

**Notes:** Greek population was based on the *2021 Greek Population and Housing Census* (2021) and on the https://www.populationpyramid.net/greece/2030/ (2030), assessed on 18 December 2024; the number of people with T2D in 2021 and in 2030 was calculated with a prevalence equal to 11.4%, according to the *IDF Diabetes Atlas 2025*; the number of people with T2D in 2030, after considering the aging effect was calculated according to the age-stratified prevalence of T2D based on the *IDF Diabetes Atlas 2025*; the distribution of patients into lines of treatment was based on Lee et al. [29]; the cost of each treatment line for T2D was retrieved from the Greek Positive List, published by the Greek Ministry of Health, which contains all drugs reimbursed by the Greek Government. Due to the complexity of treatment protocols and the large number of treatment choices per line, calculations considered the medications with the minimum monthly cost reimbursed by the Greek Social Security Fund (SSF); detailed treatment protocols and cost calculations for each treatment line are provided in Supplementary Table S1, which is based on the Therapeutic Prescription Protocol of the Greek Ministry of Health and the official Greek list of prescription drugs covered by the SSF; an inflation rate of 4.2% was considered in the 30-day cost per line of treatment per patient between the years 2021 and 2030; $\mathrm{Total}\;\mathrm{annual}\;\cos t=\sum_{i=1}^{4}\left[\left(\mathrm{Number}\;\mathrm{of}\;\mathrm{patients}\;\mathrm{in}\;\mathrm{line}\;i\right)\times \left(\text{30-day}\;\cos t\;\mathrm{for}\;\mathrm{line}\;i\right)\times 12\right]$.

**Table 3:** Estimate of total annual cost of T2D-related treatment in 2021 and 2030, with and without considering the aging effect, based on the prevalence rate estimated by the random-effects meta-analysis.

Greek Population in 2021	10,482,487	Greek Population in 2030	9,721,983	Greek Population in 2030	9,721,983
**Number of People with T2D**	**857,467**	**Number of People with Type T2D**	**795,258**	**Number of People with T2D, After Considering the Aging Effect**	**1,241,837**
**Distribution of patients into line of treatments**		**Distribution of patients into line of treatments**		**Distribution of patients into line of treatments**	
First line	31%	First line	31%	First line	31%
Second line	45%	Second line	45%	Second line	45%
Third line	23%	Third line	23%	Third line	23%
Fourth line	1%	Fourth line	1%	Fourth line	1%
**Number of patients in each line of treatment**		**Number of patients in each line of treatment**		**Number of patients in each line of treatment**	
First line	265,815	First line	246,530	First line	384,969
Second line	385,860	Second line	357,866	Second line	558,827
Third line	197,218	Third line	182,909	Third line	285,623
Fourth line	8575	Fourth line	7953	Fourth line	12,418
**30-day cost for each line of treatment**		**30-day cost for each line of treatment**		**30-day cost for each line of treatment**	
First line	EUR 330,940	First line	EUR 319,207	First line	EUR 498,458
Second line	EUR 10,967,152	Second line	EUR 10,578,349	Second line	EUR 16,518,660
Third line	EUR 10,965,368	Third line	EUR 10,576,575	Third line	EUR 16,515,935
Fourth line	EUR 748,555	Fourth line	EUR 722,027	Fourth line	EUR 1,127,390
**Total annual cost for diabetes-related treatment lines**	**EUR 255,975,189.49**	**Total annual cost for diabetes-related treatment lines**	**EUR 266,353,899.84**	**Total annual cost for diabetes-related treatment lines**	**EUR 415,925,321**

**Notes:** Greek population was based on the *2021 Greek Population and Housing Census* (2021) and on the https://www.populationpyramid.net/greece/2030/ (2030), assessed on 18 December 2024; the number of people with T2D in 2021 and in 2030 was calculated with a prevalence equal to 11.4%, according to the *IDF Diabetes Atlas 2025*; the number of people with T2D in 2030, after considering the aging effect was calculated according to the age-stratified prevalence of T2D based on the *IDF Diabetes Atlas 2025*; the distribution of patients into lines of treatment was based on Lee et al. [29]; the cost of each treatment line for T2D was retrieved from the Greek Positive List, published by the Greek Ministry of Health, which contains all drugs reimbursed by the Greek Government. Due to the complexity of treatment protocols and the large number of treatment choices per line, calculations considered the medications with the minimum monthly cost reimbursed by the Greek Social Security Fund (SSF); detailed treatment protocols and cost calculations for each treatment line are provided in Supplementary Table S1, which is based on the Therapeutic Prescription Protocol of the Greek Ministry of Health and the official Greek list of prescription drugs covered by the SSF; an inflation rate of 4.2% was considered in the 30-day cost per line of treatment per patient between the years 2021 and 2030; $\mathrm{Total}\;\mathrm{annual}\;\cos t=\sum_{i=1}^{4}\left[\left(\mathrm{Number}\;\mathrm{of}\;\mathrm{patients}\;\mathrm{in}\;\mathrm{line}\;i\right)\times \left(\text{30-day}\;\cos t\;\mathrm{for}\;\mathrm{line}\;i\right)\times 12\right]$.

**Table 4:** Estimate of total annual cost of T2D-related complications, as well as of medical examinations for monitoring T2D patients in 2021 and 2030, with and without considering the aging effect, based on the prevalence rate estimated by the GBD project.

		2021	2030	2030 *
Number of people with T2D		1,195,004	1,108,306	1,241,837
Number of T2D patients with complications		167,355	155,213	174,603
Estimated number of T2D patients with recommended frequency of examinations		597,502	554,153	620,919
T2D-related complications	Cost per T2D-related complication	Total annual cost of T2D-related complications		
Diabetic complications with devastating comorbidities/complications	EUR 6202.00	EUR 507,957,079	EUR 489,947,893	EUR 551,152,722
Diabetic complications without catastrophic comorbidities/complications	EUR 2801.00			
Endoscopic or exploratory procedures for metabolic dysfunctions on the same day	EUR 297.00			
Endoscopic or exploratory procedures for metabolic dysfunctions with devastating comorbidities/complications	EUR 4296.00			
Endoscopic or exploratory procedures for metabolic dysfunctions without catastrophic comorbidities/complications	EUR 1580.00			
T2D-related medical examinations for monitoring	Cost per T2D-related medical examination for monitoring	Total annual cost of T2D-related medical examinations for monitoring		
SGPT-SGOT levels (2 times/year)	EUR 6.98	EUR 397,517,920	EUR 383,425,132	EUR 429,620,961
HbA1c levels (4 times/year)	EUR 24.64			
Determination of blood sugar/glucose (GL) (2 times/year)	EUR 4.00			
Levels of sodium, potassium and phosphorus in blood (2 times/year)	EUR 8.00			
General blood test (2 times/year)	EUR 5.76			
Creatinine levels in blood (2 times/year)	EUR 7.60			
Triglycerides levels in blood (4 times/year)	EUR 13.96			
Cholesterol levels in blood (4 times/year)	EUR 8.80			
Urea levels in blood (2 times/year)	EUR 4.00			
HDL-cholesterol levels in blood (4 times/year)	EUR 16.00			
Uric acid levels in blood (2 times/year)	EUR 4.00			
LDL-cholesterol levels in blood (4 times/year)	EUR 16.00			
General urea test (2 times/year)	EUR 3.52			
gGt levels in blood (2 times/year)	EUR 8.00			
TKE erythrocyte sedimentation rate blood test (2 times/year)	EUR 3.52			
CPK levels in blood (2 times/year)	EUR 10.04			
ALP levels in blood (2 times/year)	EUR 8.00			
Calcium levels in blood (2 times/year)	EUR 6.00			
TSH levels in blood (2 times/year)	EUR 22.00			
Urine culture (4 times/year)	EUR 20.88			
Free thyroxine (FT4) (2 times/year)	EUR 24.00			
Ultrasound (u/s) of upper abdomen (liver, which includes gallbladder, pancreas, spleen) (3 times/year)	EUR 62.70			
Doppler spectrum ultrasound and color flow imaging (once per year)	EUR 85.00			
Triplex-ultrasound arteriography: carotid and vertebral arteries in color (once per year)	EUR 60.00			
Insulin levels (Once per year)	EUR 12.38			
Triplex-Color ultrasound arteriography of the lower extremities (once per year)	EUR 44.00			
Free thyroxine (FT3) (2 times/year)	EUR 24.00			
Ultrasound (u/s) of kidneys, ureters, and bladder, prostate—males (2 times/year)	EUR 12.62			
Ultrasound (u/s) of thyroid gland/parathyroid (once per year)	EUR 8.28			
C-peptide levels (2 times/year)	EUR 19.02			
Ultrasound (u/s) of kidneys, ureters, and bladder, uterus, ovaries, fallopian tubes—females (2 times/year)	EUR 16.56			
Triplex-color aortic ultrasound arteriography (ascending aorta and aortic arch) (once per year)	EUR 44.00			
Bottomoscopy with a GOLDMAN tricopter contact lens (once per year)	EUR 7.04			
Triplex-color ultrasound arteriography of iliac arteries (once per year)	EUR 44.00			
Total annual cost of T2D-related complications and T2D-related medical examinations for monitoring		EUR 905,475,000	EUR 873,373,025	EUR 980,773,683

**Notes:** * considering the aging effect. The number of people with T2D in 2021 and number of people with T2D in 2030 were calculated with a T2D prevalence equal to 11.4%, according to the *IDF Diabetes Atlas 2025*; the number of people with type 2 diabetes in 2030, after considering the aging effect was calculated according to the age-stratified prevalence of T2D based on the *IDF Diabetes Atlas 2025*; the percent of T2D patients with complications was based on the study of Desphande et al. [30], according to which on average 14.06% of diabetic patients suffer from diabetic-related complications; the final annual cost of T2D-related complications was estimated as (Number of T2D patients with complications) × (Average cost per complication); the calculation of the final annual cost of T2D-related medical examinations was based on the hypothesis that half of the T2D patients comply with the guidelines of the Greek Ministry of Health, as regards the frequency with which T2D patients should be referred to the relevant medical examinations for monitoring; the final annual cost of T2D-related medical examinations was estimated as (Number of T2D patients complying with the guidelines of the Greek Ministry of Health) × (Sum of the annual cost/medical examination); the costs of medical procedures for T2D-related complications were based on the DRG system, as retrieved from the DRG-matching application (http://kenicd.e-healthnet.gr), assessed on 18 December 2024, provided by the Medical Society of Athens, in partnership with the Onassis Cardiac Surgery Center and the Greek Ministry of Health. Only costs reimbursed by the Greek Social Security Fund (SSF) were included; protocols for monitoring T2D patients were based on guidelines from the General Secretariat of the Greek Ministry of Health (Ministerial Decision No. Γ3γ/40426, Government Gazette B’ 2221/18.07.2016). The costs of medical examinations were retrieved from the relevant regulations and decisions of the Greek Ministry of Health and Social Solidarity. It was assumed that 50% of patients complied with the recommended monitoring frequency; total annual cost: the sum of the annual cost of T2D-related complications and the annual cost of T2D-related medical examinations for monitoring; an inflation rate of 4.2% was applied to costs between 2021 and 2030.

**Table 5:** Estimate of total annual cost of T2D-related complications, as well as of medical examinations for monitoring T2D patients in 2021 and 2030, with and without considering the aging effect, based on the prevalence rate estimated by the random-effects meta-analysis.

		2021	2030	2030 *
Number of people with T2D		857,467	795,258	1,241,837
Number of T2D patients with complications		120,560	111,813	174,603
Estimated number of T2D patients with recommended frequency of examinations		428,734	397,629	620,919
T2D-related complications	Cost per T2D-related complication	Total annual cost of T2D-related complications		
Diabetic complications with devastating comorbidities/complications	EUR 6202.00	EUR 365,923,474	EUR 352,950,771	EUR 551,152,722
Diabetic complications without catastrophic comorbidities/complications	EUR 2801.00			
Endoscopic or exploratory procedures for metabolic dysfunctions on the same day	EUR 297.00			
Endoscopic or exploratory procedures for metabolic dysfunctions with devastating comorbidities/complications	EUR 4296.00			
Endoscopic or exploratory procedures for metabolic dysfunctions without catastrophic comorbidities/complications	EUR 1580.00			
T2D-related medical examinations for monitoring	Cost per T2D-related medical examination for monitoring	Total annual cost of T2D-related medical examinations for monitoring		
SGPT-SGOT levels (2 times/year)	EUR 6.98	EUR 285,236,543	EUR 275,124,349	EUR 429,620,961
HbA1c levels (4 times/year)	EUR 24.64			
Determination of blood sugar/glucose (GL) (2 times/year)	EUR 4.00			
Levels of sodium, potassium and phosphorus in blood (2 times/year)	EUR 8.00			
General blood test (2 times/year)	EUR 5.76			
Creatinine levels in blood (2 times/year)	EUR 7.60			
Triglycerides levels in blood (4 times/year)	EUR 13.96			
Cholesterol levels in blood (4 times/year)	EUR 8.80			
Urea levels in blood (2 times/year)	EUR 4.00			
HDL-cholesterol levels in blood (4 times/year)	EUR 16.00			
Uric acid levels in blood (2 times/year)	EUR 4.00			
LDL-cholesterol levels in blood (4 times/year)	EUR 16.00			
General urea test (2 times/year)	EUR 3.52			
gGt levels in blood (2 times/year)	EUR 8.00			
TKE erythrocyte sedimentation rate blood test (2 times/year)	EUR 3.52			
CPK levels in blood (2 times/year)	EUR 10.04			
ALP levels in blood (2 times/year)	EUR 8.00			
Calcium levels in blood (2 times/year)	EUR 6.00			
TSH levels in blood (2 times/year)	EUR 22.00			
Urine culture (4 times/year)	EUR 20.88			
Free thyroxine (FT4) (2 times/year)	EUR 24.00			
Ultrasound (u/s) of upper abdomen (liver, which includes gallbladder, pancreas, spleen) (3 times/year)	EUR 62.70			
Doppler spectrum ultrasound and color flow imaging (once per year)	EUR 85.00			
Triplex-Ultrasound arteriography: carotid and vertebral arteries in color (once per year)	EUR 60.00			
Insulin levels (Once per year)	EUR 12.38			
Triplex-color ultrasound arteriography of the lower extremities (once per year)	EUR 44.00			
Free thyroxine (FT3) (2 times/year)	EUR 24.00			
Ultrasound (u/s) of kidneys, ureters, and bladder, prostate—males (2 times/year)	EUR 12.62			
Ultrasound (u/s) of thyroid gland/parathyroid (once per year)	EUR 8.28			
C-peptide levels (2 times/year)	EUR 19.02			
Ultrasound (u/s) of kidneys, ureters, and bladder, uterus, ovaries, fallopian tubes—females (2 times/year)	EUR 16.56			
Triplex-Color aortic ultrasound arteriography (ascending aorta and aortic arch) (once per year)	EUR 44.00			
Bottomoscopy with a GOLDMAN tricopter contact lens (once per year)	EUR 7.04			
Triplex-color ultrasound arteriography of iliac arteries (once per year)	EUR 44.00			
Total annual cost of T2D-related complications and T2D-related medical examinations for monitoring		EUR 651,160,013	EUR 628,075,120	EUR 980,773,683

**Notes:** * considering the aging effect. The number of people with T2D in 2021 and number of people with T2D in 2030 were calculated with a T2D prevalence equal to 11.4%, according to the *IDF Diabetes Atlas 2025*; the number of people with type 2 diabetes in 2030, after considering the aging effect, was calculated according to the age-stratified prevalence of T2D based on the *IDF Diabetes Atlas 2025*; the percent of T2D patients with complications was based on the study of Desphande et al. [30], according to which, on average, 14.06% of diabetic patients suffer from diabetic-related complications; the final annual cost of T2D-related complications was estimated as (Number of T2D patients with complications) × (Average cost per complication); the calculation of the final annual cost of T2D-related medical examinations was based on the hypothesis that half of the T2D patients comply with the guidelines of the Greek Ministry of Health, as regards the frequency with which T2D patients should be referred to the relevant medical examinations for monitoring; the final annual cost of T2D-related medical examinations was estimated as (Number of T2D patients complying with the guidelines of the Greek Ministry of Health) × (Sum of the annual cost/medical examination); the costs of medical procedures for T2D-related complications were based on the DRG system, as retrieved from the DRG-matching application (http://kenicd.e-healthnet.gr), assessed on 18 December 2024, provided by the Medical Society of Athens, in partnership with the Onassis Cardiac Surgery Center and the Greek Ministry of Health. Only costs reimbursed by the Greek Social Security Fund (SSF) were included; protocols for monitoring T2D patients were based on guidelines from the General Secretariat of the Greek Ministry of Health (Ministerial Decision No. Γ3γ/40426, Government Gazette B’ 2221/18.07.2016). The costs of medical examinations were retrieved from the relevant regulations and decisions of the Greek Ministry of Health and Social Solidarity. It was assumed that 50% of patients complied with the recommended monitoring frequency; total annual cost: the sum of the annual cost of T2D-related complications and the annual cost of T2D-related medical examinations for monitoring; An inflation rate of 4.2% was applied to costs between 2021 and 2030.

## Data Availability

The raw data supporting the conclusions of this article will be made available by the authors on request.

## Supplementary Materials

The following supporting information can be downloaded at: https://www.mdpi.com/article/10.3390/jmahp13040053/s1: Table S1: Treatment lines for type 2 diabetes mellitus according to the Therapeutic Prescription Protocol of the Greek Ministry of Health and cost of each treatment line according to the Greek list of prescription drugs covered by the Greek Social Security (SS).
