# Supplementary material for: A Data-Driven Approach for Estimating Type 2 Diabetes-Related Costs in Greece
Source: J Mark Access Health Policy. 2025 Oct 15;13(4):53. doi: 10.3390/jmahp13040053 (PMC12551024; doi:10.3390/jmahp13040053)
Supplement: Supplementary file 1 [file jmahp-13-00053-s001.zip › jmahp-3582314-supplementary.pdf]

Supplementary Table S1. Treatment lines for Type 2 diabetes mellitus according to the Therapeutic Prescription Protocol of the Greek Ministry of Health and cost of each treatment line according to the Greek list of prescription drugs covered by the Greek Social Security (SS)

Line of treatment	Type of treatment	Name of drug being reimbursed by Greek Social Security (SS)	30- day cost according to Greek list of prescription drugs covered by SS	Minimum 30-day cost of line of treatment
**First- line treatment**				
	**Metformin**			**1.25 €**
		Glucophage	1.25 €	
		Retaform	2.27 €	
		Glucofree	1.25 €	
		Glucoplus	1.64 €	
		Glucostop	5.27 €	
		Glucoformin	11.73 €	
		Metformin/MYLAN	1.42 €	
		Glycofren	1.28 €	
**Final cost of first line of treatment**				**1.25 €**
**Second- line treatment (Metformin + ...)**				
	**Sulfonylureas (SU)**			**1.08 €**
		DEROCTYL	7.14 €	
		DIAMICRON M	1.38 €	
		GLICLAZIDE/M	1.38 €	
		GLICRON	3.64 €	
		DAONIL	1.55 €	
		ADINSULIN-S	1.49 €	
		ANOVIS	1.49 €	
		DIA-BAN	1.49 €	
		DIALOSA	1.49 €	
		GLIDIL	1.49 €	
		GLIMEPIRION	1.08 €	
		GLIMERID	1.08 €	
		GLIMEXIN	1.08 €	
		GLIPERIN	1.08 €	
		MEPIRID	1.49 €	
		RALTONE	1.08 €	
		SOLOSA	1.27 €	
		SUCRYL	1.48 €	
		TETIG	1.16 €	
	**Thiazolidinedione**			**7.98 €**
		ACTOS	8.19 €	
		GLITACT	8.24 €	
		GLIZON	7.98 €	
		PIOGLITAZONE	8.24 €	
		RAGLITAN	8.24 €	
		ZIPION	8.24 €	
		SAHAR	8.89 €	
	**Dipeptidyl Peptidase-4 inhibitors (DPP-4)**			**19.18 €**
		JANUVIA	26.52 €	
		XELEVIA	37.64 €	
		GALVUS	22.98 €	
		JALRA	22.98 €	
		ONGLYZA	37.64 €	
		VIPIDIA	19.18 €	
		TRAJENTA	37.64 €	
	**Glucagon–like peptide-1 (GLP-1) receptor agonists**			**74.01 €**
	TRULICITY<br>OZEMPIC	BYETA	74.01 €	
		VICTOZA	106.64 €	
		LYXUMIA	83.52 €	
		BYDUREON	105.40 €	
	**Sodium-glucose cotransporter 2 (SGLT2) inhibitors**			**48.78 €**

			48.78 €	
		FORXIGA	48.78 €	
		INVOKANA	50.15 €	
		JARDIANCE	49.21 €	
	**Insulin**			
		ACTRAPID	12.04 €	
		HUMULIN REG	12.77 €	
		HUMALOG	19.94 €	
		FIASP	22.43 €	
		NOVORAPID	21.64 €	
		APIDRA	18.96 €	
		HUMULN NPH	13.52 €	
		PROTOPHANE	12.08 €	
		HUMULIN M3	14.68 €	**12.04 €**
		MIXTARD	12.87 €	
		HUMALOG	36.06 €	
		NOVOMIX	43.05 €	
		ABASAGLAR	89.37 €	
		LANTUS	31.88 €	
		TOIJJEO	46.57 €	
		LEVEMIR	63.39 €	
		TRESIBA	76.11 €	
		XULTOPHY	144.20 €	
**Cost of Metformin+**				
Sulfonylureas (SU)				2.32 €
Thiazolidinedione				9.23 €
Dipeptidyl Peptidase-4 inhibitors (DPP-4)				20.42 €
Glucagon–like peptide-1 (GLP-1) receptor agonists				75.26 €
Sodium-glucose cotransporter 2 (SGLT2) inhibitors				50.03 €
Insulin				13.29 €
**Final cost of second line of treatment**				**28.42 €**
**Third- line treatment (Metformin + …)**				
	**Sulfonylureas (SU) + …**			
		Thiazolidinedione		10.30 €
		Dipeptidyl Peptidase-4 inhibitors (DPP-4)		21.50 €
		Glucagon–like peptide-1 (GLP-1) receptor agonists		76.33 €
		Sodium-glucose cotransporter 2 (SGLT2) inhibitors		51.10 €
		Insulin		14.36 €
	**Thiazolidinedione + …**			
		Dipeptidyl Peptidase-4 inhibitors (DPP-4)		28.40 €
		Glucagon–like peptide-1 (GLP-1) receptor agonists		83.24 €
		Sodium-glucose cotransporter 2 (SGLT2) inhibitors		58.01 €
		Insulin		21.27 €
	**Dipeptidyl Peptidase-4 inhibitors (DPP-4) + …**			
		Glucagon–like peptide-1 (GLP-1) receptor agonists		94.43 €
		Sodium-glucose cotransporter 2 (SGLT2) inhibitors		69.21 €
		Insulin		32.46 €
	**Glucagon–like peptide-1 (GLP-1) receptor agonists + …**			
		Sodium-glucose cotransporter 2 (SGLT2) inhibitors		124.04 €
		Insulin		87.30 €
	**Sodium-glucose cotransporter 2 (SGLT2) inhibitors + …**			
		Insulin		62.07 €
**Final cost of third line of treatment**				**55.60 €**
**Fourth- line treatment (Metformin + Basic Insulin + Nutrition Insulin/ GLP-1)**				
**Final cost of fourth line of treatment**				**87.30 €**

Notes: Final cost of first line treatment was calculated as the minimum 30-day cost of each individual first treatment line; Final cost of second line treatment was calculated as the average of the individual 30-day costs of the second lines of treatment; Final cost of third line treatment was calculated as the average of the individual 30-day costs of the third lines of treatment; Final cost of fourth line of treatment was calculated as the sum of: Final cost of first line + Minimum cost of insulin treatment available in the Greek market + Minimum cost of GLP-1 treatments available in the Greek market
